# Improvised Neutral Zone Technique in a Completely Edentulous Patient with an Atrophic Mandibular Ridge and Neuromuscular Incoordination: A Clinical Tip

**DOI:** 10.7759/cureus.1189

**Published:** 2017-04-24

**Authors:** Prathibha Saravanakumar, Saravanan Thirumalai Thangarajan, Umamaheswari Mani, Anand Kumar V

**Affiliations:** 1 Department of Prosthodontics, Faculty of Dental Sciences, Sri Ramachandra University, Porur, Chennai, India

**Keywords:** neutral zone, atrophic ridge, admix material, neuromuscular incoordination

## Abstract

Resorption of mandibular ridges is a multifactorial and biomechanical disease that is chronic, progressive, irreversible, and cumulative leading to loss of sulcular depth, vertical dimension loss, and decreased lower facial height. Some common neurological, hormonal, and metabolic disorders affect the adaptability of dentures, and this can be diagnosed by a trained prosthodontist with proper history-taking and clinical examination.The denture becomes passive due to complex neuromuscular control and causes difficulties in impression-making, mastication, and swallowing, which in turn leads to loss of retention and stability in complete dentures. Hence, residual ridge resorption becomes a challenging scenario for a clinician during fabrication of complete dentures. The neutral zone concept plays a significant role in overcoming these challenges. The neutral zone is the area where the outward forces from the tongue are neutralized or nullified by the forces of the lips and cheeks acting inward during functional movements.The neutral zone technique is an alternative approach for the construction of lower complete dentures. It is most effective for dentures where there is a highly atrophic ridge and history of denture instability. The technique aims to construct a denture that is shaped by muscle function and is in harmony with the surrounding oral structures. The technique is by no means new, but it is a valuable one. It is rarely used because of the extra clinical step involved and its complexity. Complete and partial denture failures are often related to non-compliance with neutral zone factors. Thus, the evaluation of the neutral zone is an important factor. Increased retention and stability with reduced chairside time are the salient features of this new approach to any clinically challenging situation in complete dentures.This clinical report describes a modification of the conventional neutral zone technique using improvised procedures to minimize chairside visits for a patient with an atrophic mandibular ridge and neuromuscular incoordination.

## Introduction

The principal concern for all patients is to retain all of their teeth comfortably throughout their lives through good oral health. Once a patient loses teeth, rejuvenating oral function and maintaining harmony with the muscles of the temporomandibular joints and the stomatognathic system is crucial. As the life expectancy of the population has increased, there is also a proportional increase in the complexity of complete denture cases. The unstable mandibular complete denture is a fundamental yet challenging scenario for a prosthodontist. Residual ridge resorption (RRR) is a chronic, progressive, irreversible, and disabling disease, probably of multifactorial origin [1]. RRR is an inevitable and natural physiologic process [2-4]. The neutral zone technique is favorable for patients with multitudinous, unstable, unretentive mandibular complete dentures. The goal of this technique is to place the teeth such that the forces exerted by the tongue and the cheek muscles are nullified, and the teeth remain in a safe, protected zone. Traditionally, the arrangement of teeth is based on the principles of teeth-setting. However, in the neutral zone technique, the placement of teeth is dictated by the oral musculature that varies from one patient to another. Various materials such as impression compounds [5], tissue conditioners [6], waxes [7], and impression plaster [8] have been used for recording the neutral zone, and each material has its inherent advantages and disadvantages. In this clinical technique, admix material was used to record the neutral zone in a patient with a neuromuscular disorder. The primary and the secondary impressions were made during the first clinical visit, and the jaw relation procedure and the neutral zone were recorded in the second clinical visit. This technique reduced the chairside time and the number of appointments or visits.

## Case presentation

**Figure 1:**
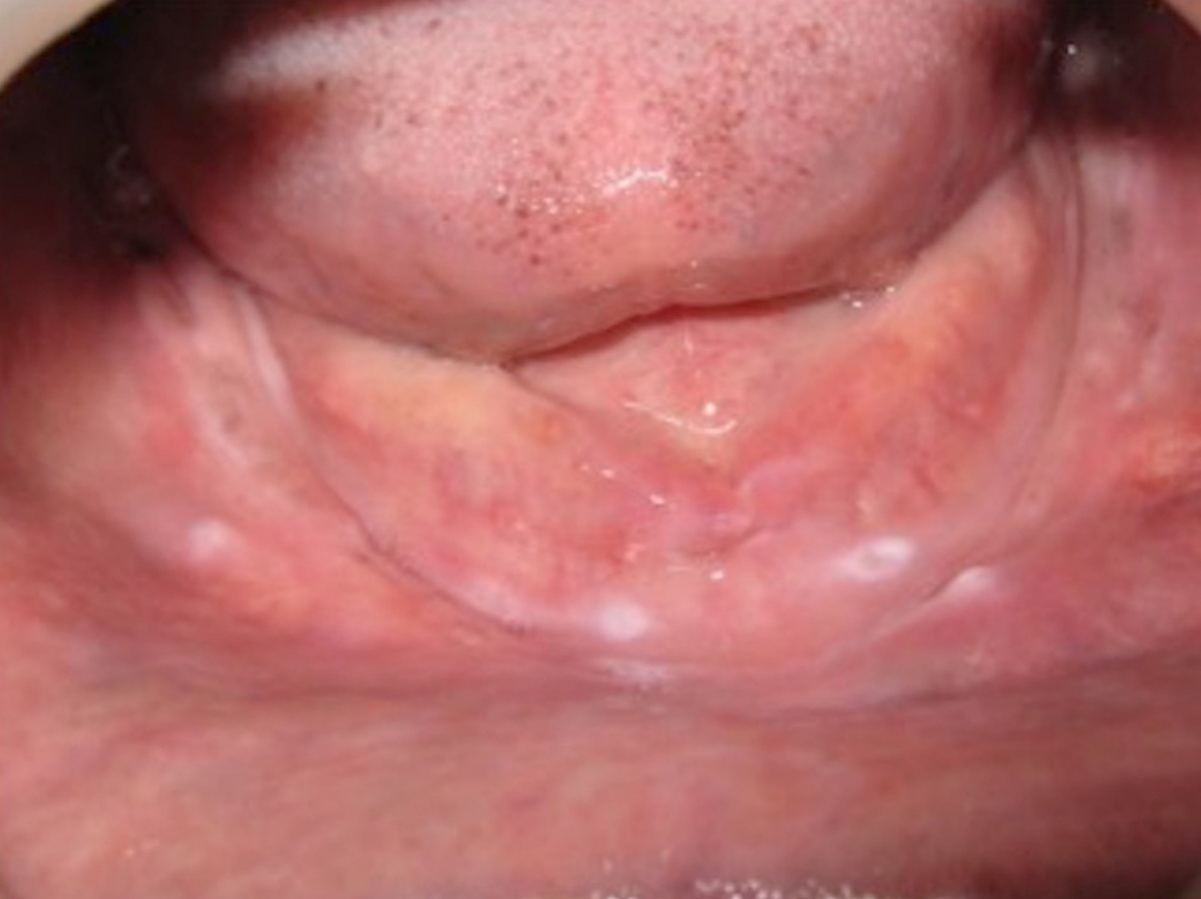
Highly Resorbed Mandibular Ridge

A 64-year-old man reported to the Department of Prosthodontics, Faculty of Dental Sciences, Sri Ramachandra University, Chennai, India, with the chief complaint of an unstable loose mandibular denture. The medical history of the patient revealed that he was diabetic, hypertensive, and under medication. The patient presented with a history of neuromuscular incoordination for the past four years. He also complained of difficulty moving his jaws, normally being a complete denture wearer for the past seven years, leading to difficulty in chewing and speech, primarily due to loose lower dentures. His past medical history was found relevant for this case report. Manipulation with removable dentures, particularly with complete ones, is based on a very complex pattern of neuromuscular coordination. The denture, in itself a lifeless and passive implement, is completely useless unless operated by the neuromusculature. Diabetes mellitus is known to produce tenderness of the mucosa, rendering it prone to infections and dryness of the oral mucosa and glossodynia. The patients will complain of a burning sensation beneath the dentures, which they usually attribute to the dentures and futilely try to relieve the symptoms by making new ones. On clinical examination, the maxillary residual alveolar ridge was rounded and well formed, but the mandibular residual ridge was unfavorable due to a high degree of resorption (classified as Atwood’s Order V - low and well-rounded) [2] (Figure 1).

 

The treatment approach for this patient was to construct a mandibular denture using the conventional neutral zone technique and to use improvised procedures to minimize the chairside visits for the patient.

### Objectives of the treatment

**Figure 2:**
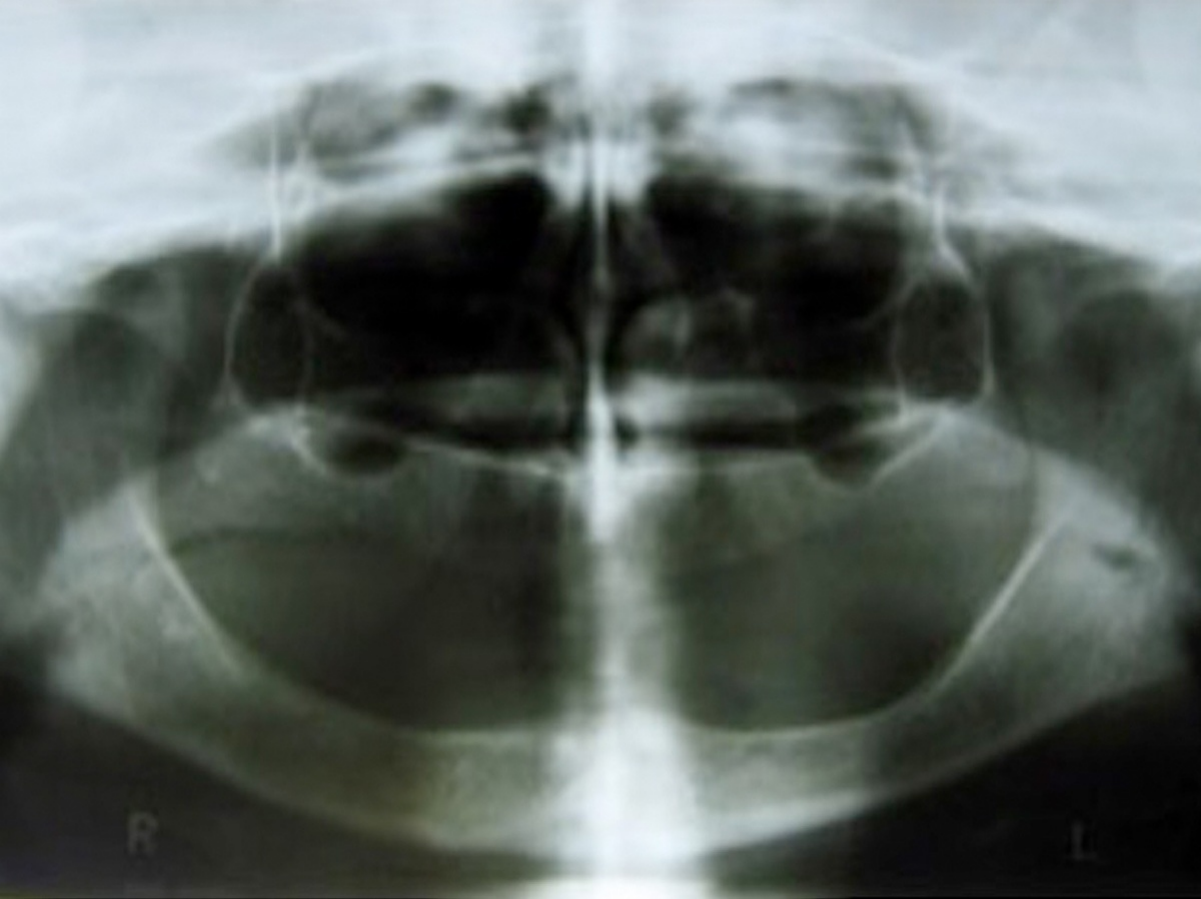
Orthopantomograph

The objectives of the treatment are rehabilitation with complete denture therapy in a patient with poor neuromuscular coordination using an improvised neutral zone technique to achieve maximum prosthesis stability, comfort, and function; locating the neutral zone and arranging the denture teeth accordingly; and minimizing the ongoing diminution of the residual alveolar ridges. Figure 2 reveals an orthopantograph of a severely resorbed mandibular arch.

The orofacial musculature plays a substantial role in developing the external polished surface of the denture and teeth arrangement. Forces developed during various muscular functions like chewing, speaking, and swallowing vary in direction and magnitude in each individual and in dissonant periods of time [9]. This is particularly notable in patients with neuromuscular incoordination, which is recorded by the neutral zone impression. 

Clinical Visit 1

**Figure 3:**
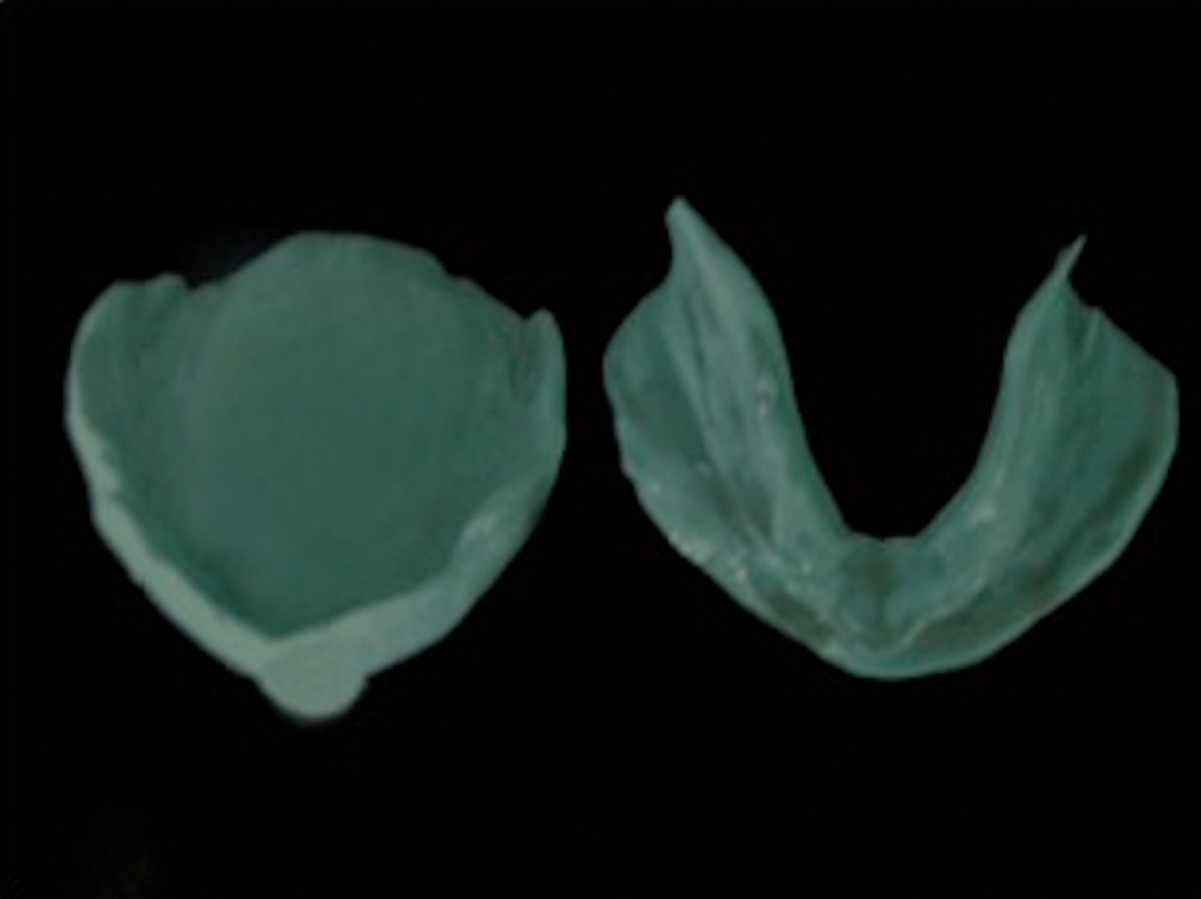
Secondary impression Secondary impression of maxillary and mandibular ridges with admix material

During the patient’s first visit, as described in the conventional neutral zone technique, a preliminary impression of the maxillary and mandibular edentulous residual ridges was made with irreversible hydrocolloid impression material (Zhermack Dust-free Thixotropic Tropicalgin, Zhermack SpA, Badia Polesine [RO], Italy). The impressions were immediately cast in dental plaster (Bombay Burmah Trading Corporation, Ltd., Mumbai, India), and primary casts were prepared. Custom trays were fabricated with DPI (Dental Products of India) - RR cold cure acrylic material (Bombay Burmah Trading Corporation, Ltd., Mumbai, India). On the same day, border molding was done with admix material – three parts by weight of impression compound and seven parts by weight of tracing compound (DPI - Pinnacle Impression Compound and Tracing Sticks, The Bombay Burmah Trading Corporation, Ltd., Mumbai, India) [10].The secondary impression was also made with the admix material (Figure 3).

The master casts were poured in dental stone (Zhermack Elite Model Stone, Zhermack SpA, Badia Polesine [RO], Italy) and record bases were constructed with self-cure acrylic resin for the maxillary cast and heat-cure acrylic resin for the mandibular cast to improve record base stability. The wax occlusal rim was fabricated for the maxillary arch. A record base over the mandibular cast was fabricated with self-cure acrylic pillars.

Clinical Visit 2

The maxillary occlusal rim was inserted and parallelism was verified using the Fox occlusal plane. The mandibular record base was also placed in the patient’s mouth and checked for extension and stability by guiding the patient to perform mandibular movements. Once the mandibular record base was stabilized, the vertical jaw relation was determined with the help of self-cure acrylic resin vertical stops (3 mm × 8 mm) placed on either side of the mandibular canine-premolar region.

**Figure 4:**
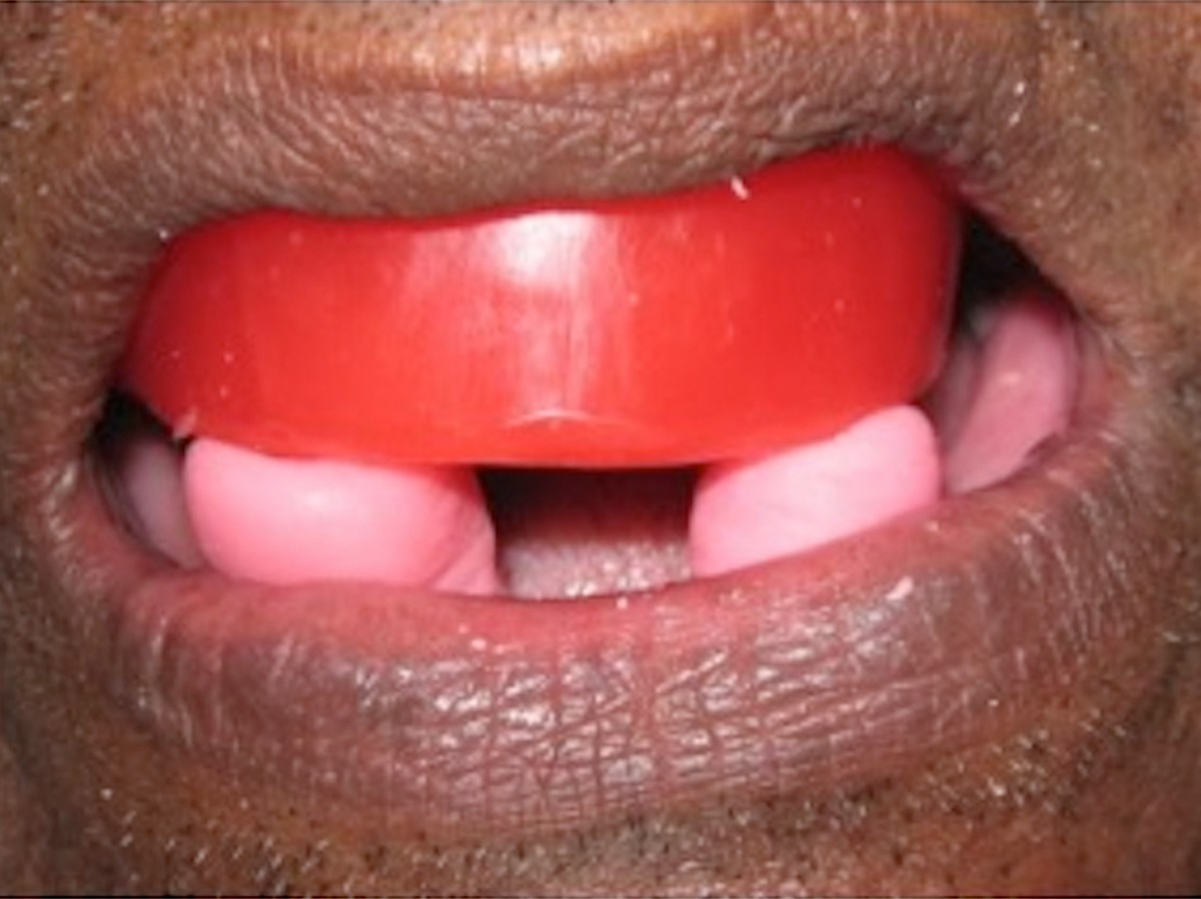
Recording Vertical Dimension Recording vertical dimension with acrylic stops

The patient was made to sit in an upright position and two prominent points were marked on the patient’s face - one on the nose and one on the chin. The vertical dimension at rest (VDR) was checked between these two points with the help of a divider and a 12-inch ruler. The determined VDR was 7.1 mm. Vertical dimension at occlusion (VDO) was determined with the help of self-cure acrylic stops fabricated on the mandibular record base. The patient was instructed to bite on the acrylic stops as it reached the early dough stage along with the maxillary occlusal rim, which was visualized and checked with the help of the divider and 12-inch ruler (Figure 4).

The established VDO was 6.8 mm. Once the resin was set, the excess resin was trimmed off, thus culminating the vertical jaw relation.

**Figure 5:**
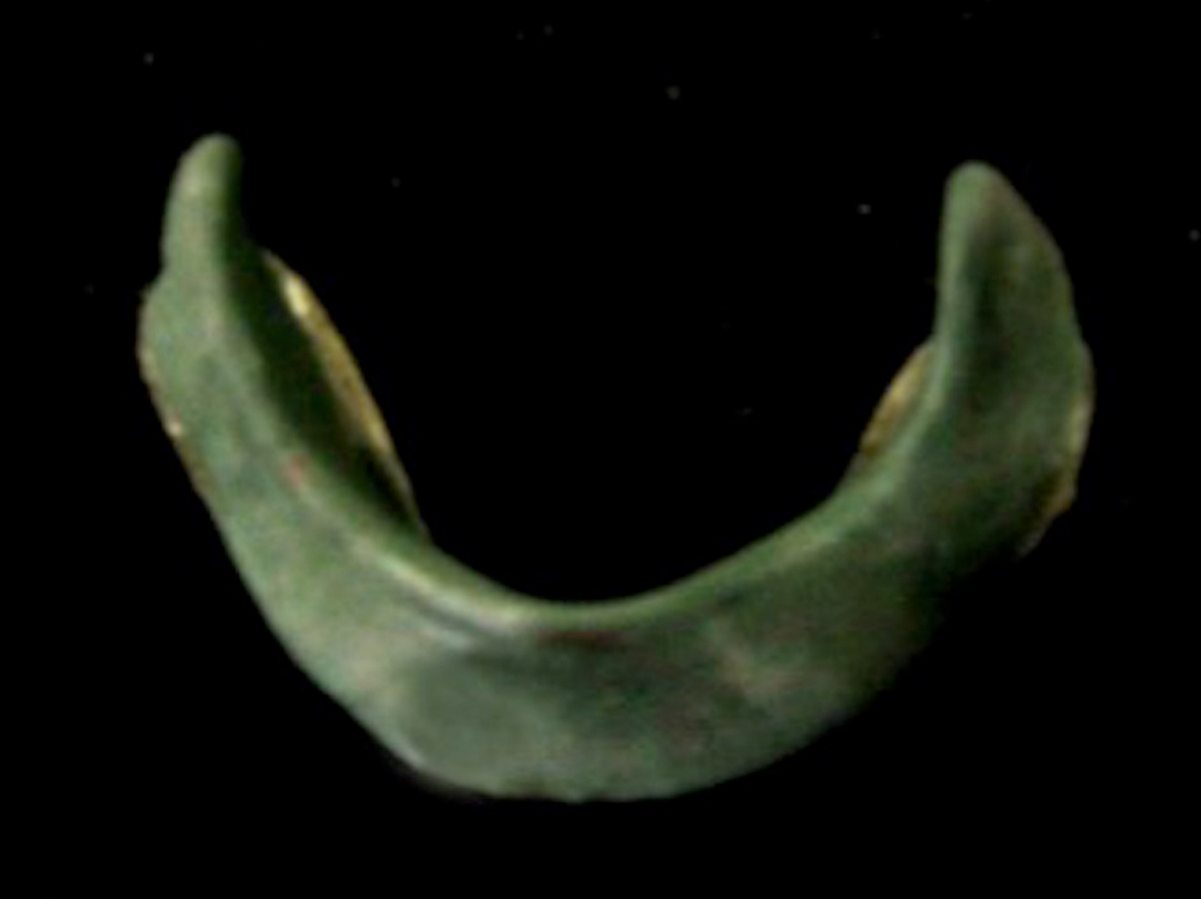
Functional movements recorded with admix material

The horizontal jaw relation was recorded using the admix material. This admix material is manipulated in the patient’s mouth at around 40° C. The patient was instructed to perform routine mandibular movements (including swallowing, sucking of the lips, and pronouncing the vowels), which aided in molding the neutral zone space (Figure 5).

 

The external surface was completely contoured by the orofacial musculature.The maxillary and the mandibular rims were fused at the centric relation.

**Figure 6:**
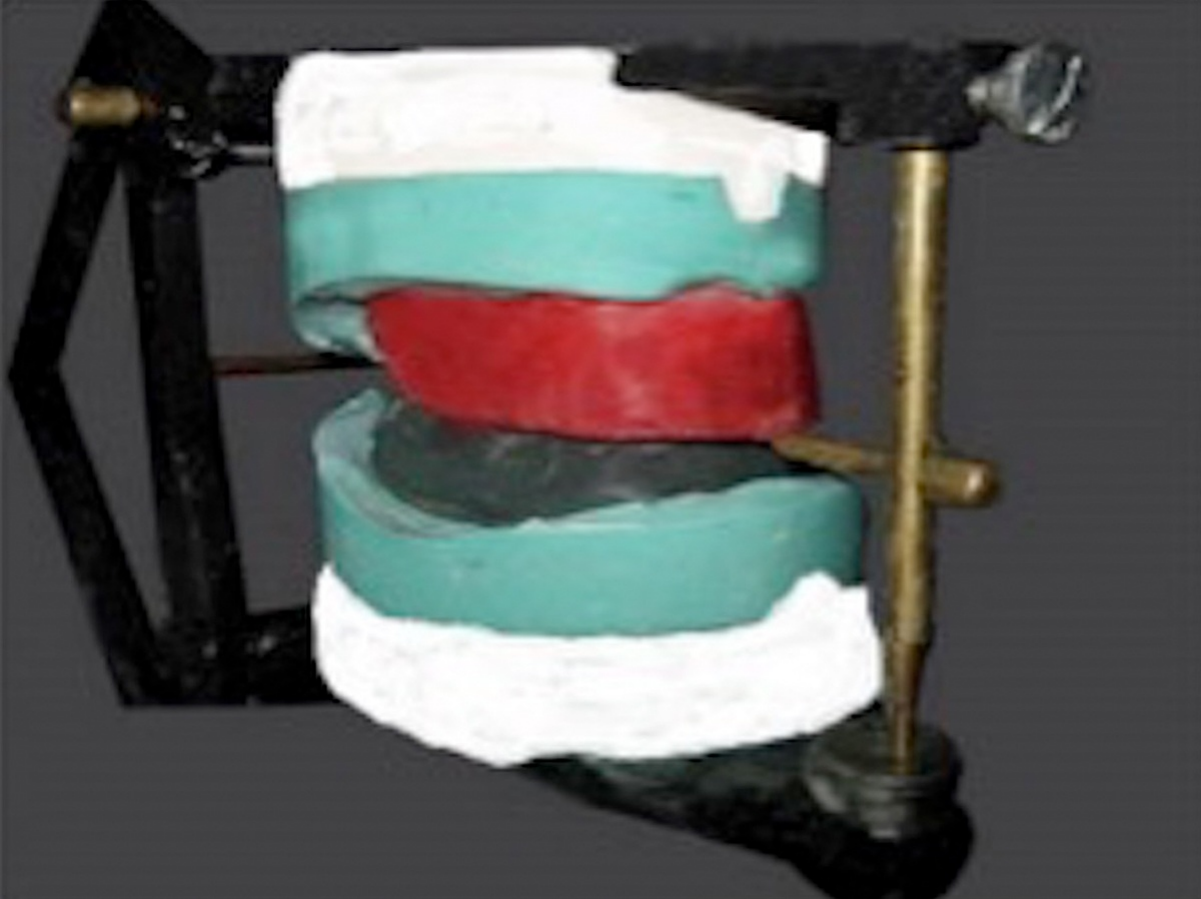
Articulated occlusal rims

The maxillary and mandibular occlusal rims were articulated in a mean value articulator to fabricate indices surrounding the neutral zone plaster impression on the mandibular cast ( Figure 6).

**Figure 7:**
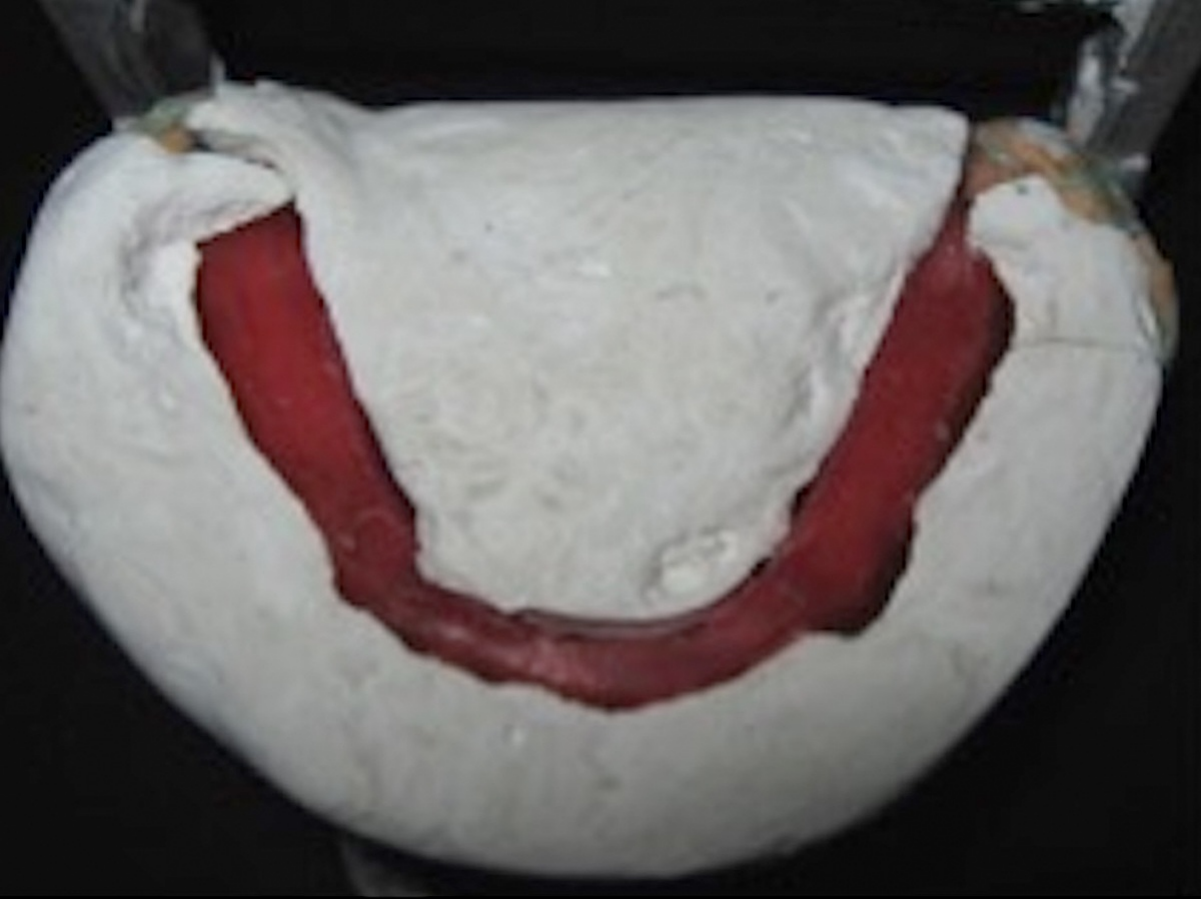
Index made with impression plaster

Three notches were made on the cast: one in the anterior and two in the posterior regions. This was followed by applying separating medium on the cast, the record base, and over the neutral zone record. Boxing was done with modeling wax, and plaster of Paris was poured into the boxing up to the upper surface. The plaster indices were sectioned into a labial and buccal index and a lingual index in order to guide the removal and placement of these indices. The neutral zone record is then removed, and the acrylic stops are trimmed off from the denture base. Separating medium was applied on the inner surfaces of the indices which were then reassembled. Wax was poured in the space representing the neutral zone, forming the new occlusal rim on the mandibular record base. Figure 7 shows the occlusal rim created and the plaster index.

The mandibular teeth were arranged following the index, and the maxillary teeth were arranged following the mandibular teeth arrangement. In order to preserve the contours established by the plaster indices in the neutral zone, no additional wax added to the denture flanges.

Clinical Visit 3

A wax try-in was performed to evaluate mandibular record base stability, aesthetics, and intraoral occlusion. The patient successfully performed all the movements mentioned earlier. The trial dentures were processed with heat-cure acrylic resin. The denture was polished so that the customized contours remained unaltered.

Clinical Visit 4

**Figure 8:**
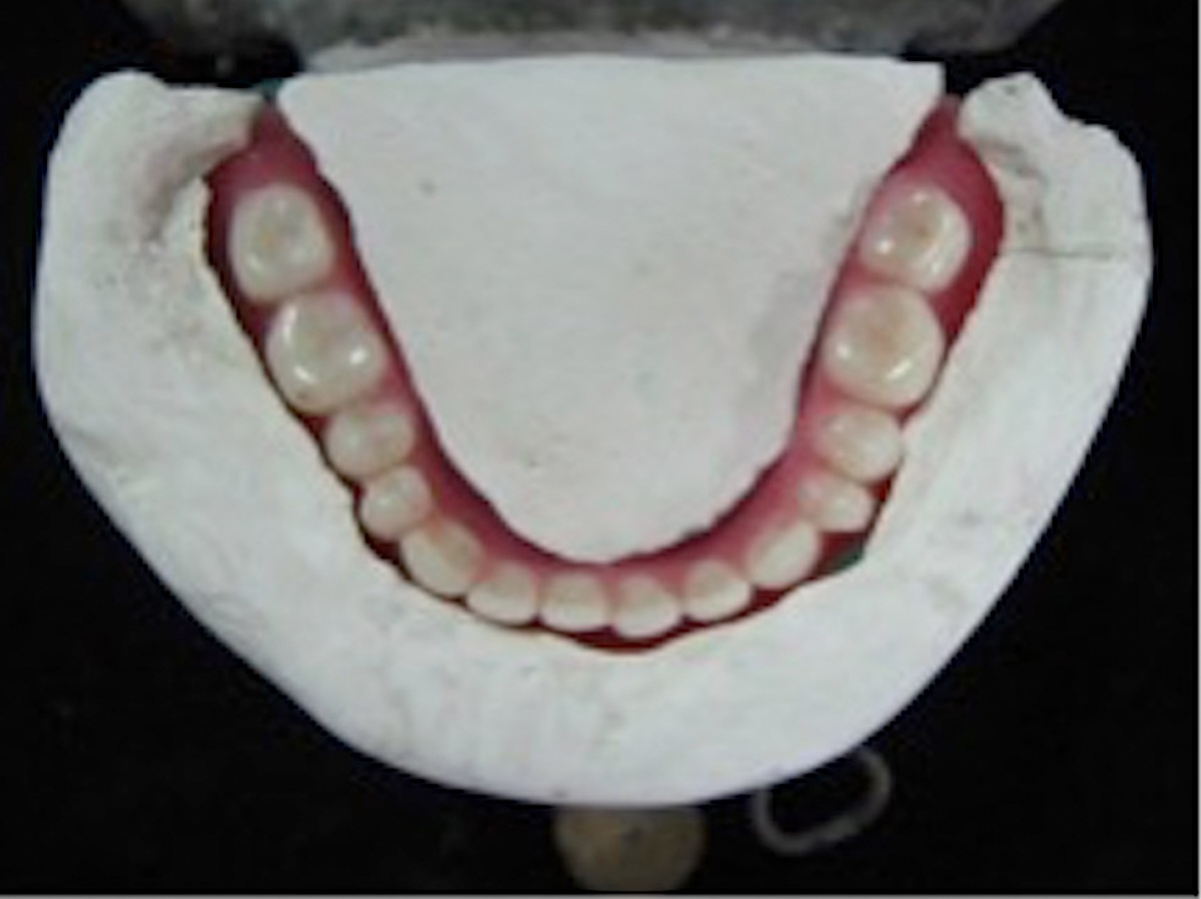
Processed denture verified with index Teeth arrangement was done in the neutral zone. The mandibular denture was processed and finished. It was verified with the plaster index prior to denture insertion.

The mandibular denture was again evaluated with the plaster index prior to denture insertion. (Figure 8)

The denture was inserted and verified for retention, stability, and occlusion. The patient was comfortable with the complete denture prosthesis. Periodic recall visits were scheduled to verify the retention, comfort, and function.

## Discussion

In the field of oral rehabilitation, particularly in geriatric prosthodontics, many factors contribute to the overall performance of complete dentures. It's a general experience that the lower denture is relatively less stable than the upper one with increasing life expectancy, age-related reduction in adaptability, and progressive severe mandibular resorption. One of the philosophies being introduced to overcome the challenge of unstable dentures in clinics is the concept of the neutral zone. The neutral zone technique was used with few modifications to achieve retention and stability in such atrophic mandibular ridges. An implant-supported over-denture is another viable treatment option but was not pursued considering the cost, duration, and the patient’s age.

The technique described in this article differs from the conventional technique by minimizing the number of patient visits and offers the added advantage of recording the physiological dynamics of oral and perioral muscle function in a simplified manner. Acrylic stops were used to judge the VDO, and the neutral zone was recorded using the swallowing technique. The mean value articulator was desirable in this case due to the patient’s medical history of neuromuscular incoordination.

Border molding was done with green stick compound, and a secondary impression was made with zinc oxide impression paste. In this case, admix material was the preferred substance for border molding and making the secondary impression as it permits the patient to mold the neutral zone with the least amount of time and effort. The admix material also helps mold the peripheral tissues, removes any soft tissue folds, and smoothes them over the mandibular bone during the impression procedure.

Over the years, a variety of materials have been used to record the neutral zone such as impression compound, impression plaster, waxes, tissue conditioners, and polyether. The impression compound material is of high viscosity, so performing oral functions such as blowing, sucking, and pursing of the lips cannot be dexterously performed. Impression plaster is chaotic and carries a risk of the patient swallowing fragments of plaster while performing functional movements. Uniform softening of the complete wax rims is critical for recording full functional movements, and if not done properly, can result in an inaccurate recording of the neutral zone. Tissue conditioners do not possess sufficient body; hence, it becomes laborious to use them even when they are supported by wire loops. Polyether impression material sets via an irreversible chemical reaction, making it difficult to perform any modification in the set material and reuse it [10].

Admix material was used for recording the neutral zone taking into consideration our patient’s history of neuromuscular incoordination (where the oral musculature could not perform its function fully). It's a combination of impression compound and green stick (low-fusing) compound in the ratio of 3:7. The mixing of a low-fusing compound with the impression compound results in a low viscosity material allowing for ease in manipulation of the oral musculature. The admix material allowed better flow and an accurate impression. The improvised technique made few modifications to the conventional technique as discussed in Table 1.

**Table 1:** Differences Between the Conventional Technique and the Improvised Neutral Zone Technique

S.no	Conventional neutral zone technique	Improvised neutral zone technique
1	Indicated for patients with resorbed mandibular ridges.	Indicated for patients with resorbed mandibular ridges and poor neuromuscular coordination.
2	Requires more clinical visits and permits the patient to mold into the neutral zone with more amount of time and effort.	Clinical visits minimized in this technique and permits the patient to mold into the neutral zone with less amount of time and effort.
3	Admix material is used for making secondary impressions. Second clinical visit required for border molding secondary impressions.	Admix material used for both border molding and the secondary impression, thus minimizing the clinical visits of the patient by making the primary and secondary impressions on the same day.
4	Facilitation of muscular control by using the conventional neutral zone technique improving the stability and control of the lower denture (by reduction of displacing forces).	Facilitation of muscular control by using the improvised neutral zone technique increasing stability and control of the lower denture (by reduction of displacing forces)for a patient with poor neuromuscular coordination.

## Conclusions

Functional and aesthetic dental treatments for patients with atrophic ridges are an inestimable service provided by a prosthodontist. This anthropoidal pouch technique is helpful in patients with atrophic ridges whose primary complaints are pain and looseness of the mandibular complete denture. The technique has proved to be efficient for patients who are not satisfied with mandibular dentures. Further, the admix material aided in recording the functions of the oral musculature in a patient with poor muscular coordination. The ability of the dental prosthesis to withstand the various forces acting on it, and the residual tissues of the ridge area (along with a properly fabricated prosthesis), help in counteracting these displacing forces and play a role in determining the success of the treatment. In this present case, all the above methods have been utilized to restore masticatory efficiency and improve comfort and aesthetics for a completely edentulous patient with an atrophic mandibular ridge and neuromuscular incoordination.
